# A53T-Alpha-Synuclein Overexpression Impairs Dopamine Signaling and Striatal Synaptic Plasticity in Old Mice

**DOI:** 10.1371/journal.pone.0011464

**Published:** 2010-07-07

**Authors:** Alexander Kurz, Kay L. Double, Isabel Lastres-Becker, Alessandro Tozzi, Michela Tantucci, Vanessa Bockhart, Michael Bonin, Moisés García-Arencibia, Silke Nuber, Falk Schlaudraff, Birgit Liss, Javier Fernández-Ruiz, Manfred Gerlach, Ullrich Wüllner, Hartmut Lüddens, Paolo Calabresi, Georg Auburger, Suzana Gispert

**Affiliations:** 1 Department of Neurology, Goethe University Medical School, Frankfurt, Germany; 2 Neuroscience Research Australia and the University of New South Wales, Sydney, Australia; 3 Clinica Neurologica, Università di Perugia, Ospedale S. Maria della Misericordia, Perugia, Italy; 4 Molecular Psychopharmacology, Department of Psychiatry, Johannes Gutenberg University, Mainz, Germany; 5 Department Medical Genetics, University of Tübingen, Tübingen, Germany; 6 Department of Biochemistry and Molecular Biology and “Centro de Investigación Biomédica en Red de Enfermedades Neurodegenerativas (CIBERNED)”, Faculty of Medicine, Complutense University, Madrid, Spain; 7 Department of Medical Genetics, University of Tübingen, Tübingen, Germany; 8 Institute of General Physiology, University of Ulm, Ulm, Germany; 9 Laboratory for Clinical Neurochemistry, Department Child and Adolescent Psychiatry, Psychosomatics and Psychotherapy, Bayerische Julius-Maximilian-Universität, Würzburg, Germany; 10 Department of Neurology, Rheinische Friedrich Wilhelms Universität, Bonn, Germany; 11 Fondazione Santa Lucia I.R.C.C.S.-C.E.R.C., European Brain Research Institute, Roma, Italy; National Institute on Aging (NIA), National Institutes of Health (NIH), United States of America

## Abstract

**Background:**

Parkinson's disease (PD), the second most frequent neurodegenerative disorder at old age, can be caused by elevated expression or the A53T missense mutation of the presynaptic protein alpha-synuclein (SNCA). PD is characterized pathologically by the preferential vulnerability of the dopaminergic nigrostriatal projection neurons.

**Methodology/Principal Findings:**

Here, we used two mouse lines overexpressing human A53T-SNCA and studied striatal dysfunction in the absence of neurodegeneration to understand early disease mechanisms. To characterize the progression, we employed young adult as well as old mice. Analysis of striatal neurotransmitter content demonstrated that dopamine (DA) levels correlated directly with the level of expression of SNCA, an observation also made in SNCA-deficient (knockout, KO) mice. However, the elevated DA levels in the striatum of old A53T-SNCA overexpressing mice may not be transmitted appropriately, in view of three observations. First, a transcriptional downregulation of the extraneural DA degradation enzyme catechol-*ortho*-methytransferase (COMT) was found. Second, an upregulation of DA receptors was detected by immunoblots and autoradiography. Third, extensive transcriptome studies via microarrays and quantitative real-time RT-PCR (qPCR) of altered transcript levels of the DA-inducible genes *Atf2*, *Cb_1_*, *Freq*, *Homer1* and *Pde7b* indicated a progressive and genotype-dependent reduction in the postsynaptic DA response. As a functional consequence, long term depression (LTD) was absent in corticostriatal slices from old transgenic mice.

**Conclusions/Significance:**

Taken together, the dysfunctional neurotransmission and impaired synaptic plasticity seen in the A53T-SNCA overexpressing mice reflect early changes within the basal ganglia prior to frank neurodegeneration. As a model of preclinical stages of PD, such insights may help to develop neuroprotective therapeutic approaches.

## Introduction

Alpha-synuclein (SNCA) is a soluble, natively unfolded 17 kDa protein, which acquires an antiparallel conformation after binding to phospholipid surfaces. Its abundant expression in neurons and the selective localization to nerve terminals point to an important role for signaling and synaptic maintenance. Although available evidence implicates SNCA in synaptic vesicle recycling and neurotransmitter release in interaction with the vesicle co-chaperone cysteine-string protein-α (CSPα) [1], [2], its detailed function is not yet understood.

SNCA missense mutations, such as A53T, as well as increased expression of wild-type (WT) SNCA result in rare forms of Parkinson's disease (PD) characterized by early-onset and autosomal dominant inheritance [3], [4]. Undefined factors trigger the aggregation of WT-SNCA within neurites and the cytoplasm of degenerating neurons to form so-called Lewy bodies and Lewy neurites which are microscopically visible in nearly all variants of PD, provoking the concept that SNCA oligomerization plays a key role in the neurodegenerative cascade [5]. While microscopic SNCA aggregates appear particularly early in the brainstem and the olfactory bulb [6], it is the preferential loss of dopamine (DA) secreting nerve terminals in the striatum and the corresponding cell bodies in the substantia nigra (SN) pars compacta [7] which underlies the deficits in spontaneous movement activity characteristic of PD patients.

Imaging studies of early stage PD have shown that the progressive loss of dopaminergic terminals and DA signaling in the striatum is accompanied by compensatory receptor hypersensitivity of the postsynaptic medium spiny neurons (MSNs) evidenced by increased DA receptor D1 and D2 (DRD1 and DRD2) levels [8].

In animal studies, striatal neurons respond to altered DA signaling with changes in second messenger levels and transcriptional activity. DA-regulated gene expression modulates postsynaptic excitability in the striatum [9] and synaptic plasticity as a correlate of learning [10], until eventually loss of MSN spines occurs [11].

In PD patients, the dynamic adaptations of striatal neurons to reduced dopaminergic innervation and to chronic pharmacological substitution of DA are likely to influence the clinical symptoms of the disease, the therapeutic benefit as well as the adverse long term effects such as dyskinesias [12].

To study the initial and progressive pathogenic effects of SNCA on intact dopaminergic terminals *in vivo*, we aged two independent transgenic mouse lines (PrPmtA and PrPmtB) overexpressing human A53T-SNCA at levels which do not result in visible protein aggregates, nor in neuronal loss within the dopaminergic nigrostriatal projection [13].

Our data indicate that dopaminergic neurotransmission is altered in several ways. Striatal DA levels were modulated by SNCA expression levels, suggesting presynaptic dysfunction in old A53T-SNCA overexpressing mice with DA elevation. Furthermore, both the transcriptional downregulation of the extraneural DA metabolizing enzyme catechol-*ortho*-methyltransferase (COMT) and the upregulation of postsynaptic DA receptors (DRD1 and DRD2) suggest that diminished amounts of DA in the extracellular space result in compensatory efforts to maximize the postsynaptic signal.

Finally, a decreased postsynaptic transcriptional response to DA was documented with extensive transcriptome microarray profiles and quantitative real-time RT-PCR (qPCR) in transgenic mice at old age, pointing to a progressive impairment of metabotropic glutamate receptor (mGluR)-induced endocannabinoid-mediated synaptic plasticity. This finding was supported by electrophysiological studies demonstrating impaired corticostriatal long term depression (LTD).

Taken together, our approach with aged mice elucidates at the molecular level how A53T-SNCA overexpression in the nigrostriatal projection may affect synaptic strength and integrity.

## Results

### No evidence of neurodegeneration, but insolubility of alpha-synuclein is detectable in the mouse mutants studied

**Figure 1:**
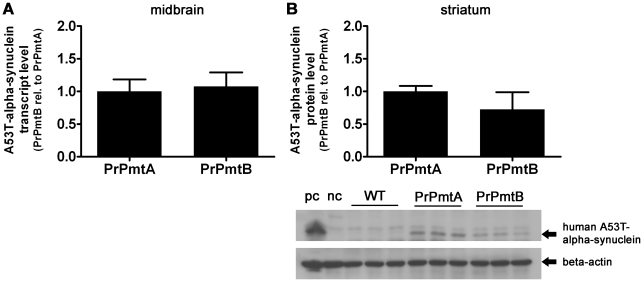
Human A53T-alpha-synuclein mRNA and protein expression at old age. Bar graphs depicting mRNA levels and protein levels of transgenic human A53T-alpha-synuclein. (A) Overexpressing mouse lines PrPmtA and PrPmtB exhibit similar mRNA levels of transgenic human A53T-alpha-synuclein in the midbrain at old age. (B) Accordingly, these mice show no differences in the protein levels of transgenic human A53T-alpha-synuclein in the striatum, the target area of the nigrostriatal projection. Data was normalized to the loading control (*Tbp* transcript or beta-actin protein) and to the mean values of PrPmtA mRNA or PrPmtA protein levels, respectively, and presented as bar graphs. N = 3 animals/genotype. “pc”  =  positive control (human brain protein medley/#635301, Clontech); “nc”  =  negative control (mouse brain extract/#sc-2253, Santa Cruz Biotechnology).

Similar transgene expression levels were documented in the nigrostriatal projection of the PrPmtA and the PrPmtB mouse line at old age, regarding the transcript levels in midbrain (Fig. 1A) and the protein levels in striatum (Fig. 1B). The expression of endogenous mouse SNCA and transgenic human A53T-SNCA in the striatum of PrPmtA, PrPmtB or KO animals was also similar at young and old age (Fig. S1). Counts of tyrosine hydroxylase (TH) positive dopaminergic neurons in consecutive sections throughout the entire SN of PrPmtA, PrPmtB or KO animals did not demonstrate any significant differences in nigral neuron numbers (data not shown). Furthermore, TH immunoblots demonstrated a similar level of dopaminergic innervation to the striatum at both ages (Fig. S1). These data are in agreement with our previous findings of a lack of neurodegeneration during the life span of our A53T-SNCA overexpressing mice [13] and suggest that these animals represent a milder form or an earlier stage of pathology compared with other SNCA-overexpressing mouse models which exhibit progressive striatal nerve terminal loss and dopaminergic axon degeneration [14], [15], [16].

In PD, the neurodegeneration correlates to the aggregation of alpha-synuclein, which can be detected microscopically or by sequential fractionation of soluble versus insoluble proteins. Although immunogold electron microscopy had been unable to demonstrate alpha-synuclein aggregates in our A53T-SNCA overexpressing mice, we now used an antibody (Mc42) specifically directed against the alpha-synuclein NAC fragment which is involved in the aggregation and analyzed the presence of insoluble alpha-synuclein species. Insoluble monomeric bands were clearly found, while insoluble higher molecular forms were faintly present in the nigrostriatal tissue of some, but not all aged PrPmtA and PrPmtB mice, similar to human PD tissue (Fig. S2). Such insoluble alpha-synuclein bands were absent from the cortex and olfactory bulb of aged animals and also from nigrostriatal tissue of young animals or old WT littermates (data not shown). Although the faint high molecular species of insoluble alpha-synuclein may be insufficient for microscopical demonstration, these data are compatible with a mild or early stage of pathogenesis.

### Striatal DA concentration correlates with SNCA expression levels in old mice

In view of previous data suggesting neuronal dysfunction rather than neurodegeneration in our A53T-SNCA transgenic mice [13], we assessed the effect of A53T-SNCA overexpression on dopaminergic neurotransmission by measuring DA steady state levels in striatal tissue homogenates using high pressure liquid chromatography (HPLC). Although a deficit of DA signaling would be expected in animals which model clinically manifest PD, significant increases in DA concentrations were found in both A53T-SNCA overexpressing mouse lines (PrPmtA: +32%, n = 5, p = 0.011; PrPmtB: +34%, n = 5, p = 0.013; HPLC method 1), compared with age-matched controls (n = 6) at old age (method 1). This observation was surprising and in contrast to a previously documented 30% reduction of striatal DA in 3 months old mice overexpressing an amino-acid 1–120 fragment of SNCA under control of the TH promoter [16] and a 29% reduction of striatal DA in 13–23 months old double mutant A30P/A53T-SNCA overexpressing mice [15].

**Figure 2:**
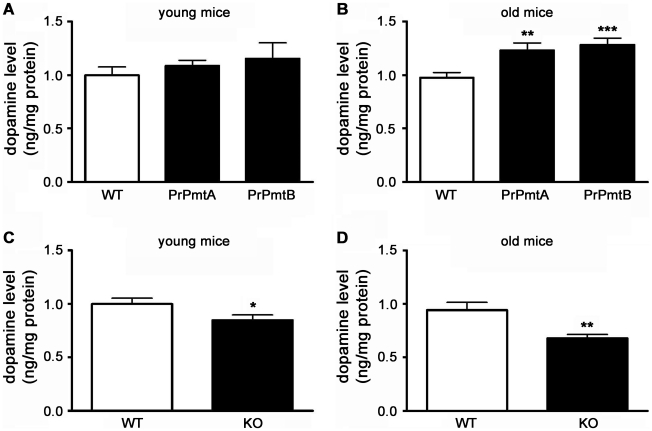
DA levels in striatal homogenates correlate to SNCA expression level. Bar graphs illustrating the dopamine levels in both A53T-SNCA overexpressing mouse lines (PrPmtA and PrPmtB), the alpha-synuclein-deficient line (KO) and corresponding wild-type (WT) animals analyzed in striatal tissue homogenates via HPLC. Increases of striatal dopamine (DA) levels were observed in young animals as a trend (A) and in old animals with high significance (B) for both A53T-SNCA overexpressing mouse lines. Correspondingly, in the SNCA-deficient (KO) mice, a smaller decrease of striatal DA levels was observed in young animals (C) which progressed in old KO animals (D). Statistical significance is reflected via asterisks (* p<0.05; ** p<0.01; *** p<0.001).

To validate our finding, additional mice were bred, dissected and assayed with a similar HPLC protocol in a different laboratory (HPLC method 2). Again, significantly enhanced DA concentrations were detected at old age (PrPmtA: +15%, n = 7, p = 0.013) while a trend towards increased striatal DA was seen in young animals (Fig. 2A and 2B). Consistent significant changes of the DA metabolites DOPAC (3,4-dihydroxyphenylacetic acid) and HVA (homovanillic acid) were not observed (data not shown). Since the DA alteration is cumulative and becomes significant only at old age (Fig. 2B), we speculate that it represents an indirect effect. Thus, A53T-SNCA overexpression appears to enhance striatal DA levels before the advent of neurodegeneration.

Conversely, analyses of old SNCA-deficient (knockout, KO) mice demonstrated a 27% reduction of striatal DA (n = 5, p = 0.012; method 1), an observation also confirmed in the independent laboratory (35% DA reduction, n = 7, p = 0.002), while young animals exhibited less reduction of striatal DA (Fig. 2C and 2D show the combined data). In studies of independent KO mouse lines, a reduction of striatal DA was also observed in young [17], [18], as well as in old [19] animals. Taken together, our findings indicate that altered striatal DA occurs in synaptic dysfunction due to SNCA gene dosage variation, not only after nerve terminal degeneration.

### Increased striatal dopamine is accompanied by reduced Comt expression

**Figure 3:**
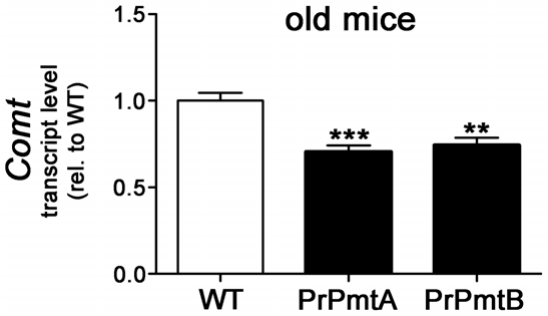
Reduced *Comt* transcript levels in striata of both transgenic lines at old age. Bar graph illustrating the reduced transcript levels for the extraneural dopamine degradation enzyme catechol-*ortho*-methyltransferase (*Comt*) in the striatum of both A53T-SNCA overexpressing mouse lines (PrPmtA and PrPmtB). Statistical significance is reflected via asterisks (** p<0.01; *** p<0.001). Expression changes were analyzed with the 2^−ΔΔCt^ method [52].

To understand whether the increased DA steady state level in the striata of old A53T-SNCA overexpressing mice is accompanied by enhanced synthesis, decreased degradation or altered compartmentalization of DA, the expression levels of different enzymes and transporters responsible for DA homeostasis were determined (Table S1). Expression changes of the dopamine transporter (*Dat*) and the vesicular monoamine transporter 2 (*Vmat2*) which were reported to correlate to DA distribution anomalies between synaptic cytosol, vesicles and the synaptic cleft [20], [21] were not detectable in our qPCR assessment of these candidate genes. However, a highly significant decrease of the transcript levels of the DA degradation enzyme COMT was observed in both A53T-SNCA overexpressing mouse lines (Fig. 3; PrPmtA: −30%, n = 6, p = 0.0005 and PrPmtB: −26%, n = 7, p = 0.0018).

In view of the extraneural localization of this enzyme, this transcriptional downregulation probably represents a response of the surrounding glia to abnormal DA levels in the extracellular space.

### Dopamine receptors DRD1 and DRD2 are upregulated in the striatum of old A53T-SNCA overexpressing mice

**Figure 4:**
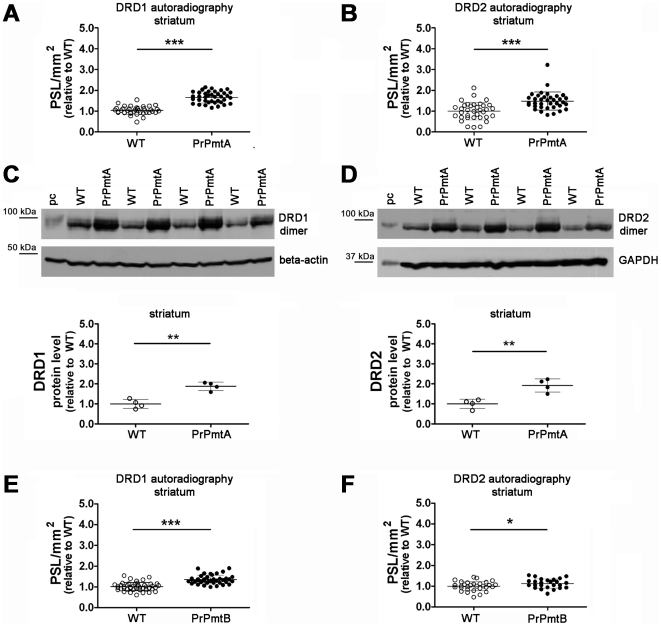
Autoradiography and immunoblot reveal elevated striatal DRD1A and DRD2 levels. Increased levels of DRD1A in lines PrPmtA (A; 1.63-fold; p<0.0001; 3 mice/genotype; slices: WT n = 39, PrPmtA n = 38) and PrPmtB (E; 1.34-fold; p<0.0001; 2 mice/genotype; slices: WT n = 44, PrPmtA n = 40), as well as increased levels of DRD2 in lines PrPmtA (B; 1.48-fold; p<0.0001; 3 mice/genotype; slices: WT n = 35, PrPmtA n = 36) and PrPmtB (F; 1.13-fold; p<0.034; 2 mice/genotype; slices: WT n = 30, PrPmtA n = 25) were observed at old age. Independent data validation was successful in immunoblot experiments employing striatal protein extracts of old PrPmtA mice for DRD1A (C; 1.88-fold; p = 0.001; WT n = 4, PrPmtA n = 4) and DRD2 (D; 1.92-fold; p = 0.004; WT n = 4, PrPmtA n = 4) normalized to the beta-actin/GAPDH loading control, while the elevation did not reach significance for PrPmtB derived samples (data not shown). Student's t-test was employed for all statistical analyses. PSL  =  photo-stimulated luminescence; pc  =  positive control (mouse brain extract/#sc-2253, Santa Cruz Biotechnology). Statistical significance is reflected via asterisks (* p<0.05; ** p<0.01; *** p<0.001).

Since *Comt* reduction seems a plausible effort to enhance DA signaling, we asked whether the increased striatal DA affects signaling in old A53T-SNCA overexpressing mice. To elucidate the postsynaptic response to DA, expression levels of the main D1-like and D2-like DA receptors in the striatum DRD1 and DRD2 were measured by two independent techniques. In the striatum of old PrPmtA mice, clearly significant upregulations of DRD1 (1.63-fold in [^3^H]-SCH23390 autoradiography, Fig. 4A; 1.88-fold in immunoblots, Fig. 4C) and of DRD2 (1.48-fold in [^3^H]-spiperone autoradiography, Fig. 4B; 1.92-fold in immunoblots, Fig. 4D) were observed, while in the striatum of PrPmtB mice of similar age, the upregulation was smaller and significant only in autoradiography (1.34-fold in [^3^H]-SCH23390 autoradiography, Fig. 4E; 1.13-fold in [^3^H]-spiperone autoradiography, Fig. 4F), but not in immunoblots with non-linear enhanced chemiluminescence detection (data not shown). Upregulation of DA receptors and postsynaptic hypersensitivity are known compensatory efforts associated with deficient DA signaling [8]. The enhanced DA receptor levels together with the downregulated *Comt* expression therefore may indicate a cellular effort to compensate abnormal DA signal transduction.

### Overexpression of A53T-SNCA changes Homer1 expression in several brain regions

It remained unclear whether the reduction of *Comt* and the upregulation of the DA receptors were sufficient to compensate the dysfunction in DA signaling. To investigate the progression of pathology in a hypothesis-free manner, extensive profiling of the transcriptome was undertaken to screen for expression changes (i) induced by the A53T-SNCA genotype in several brain regions and ages or (ii) induced progressively over time, occurring consistently and selectively only in the striatum of both transgenic lines, but not in WT striatum or any other brain region of the three mouse lines under study. All microarray data discussed in this publication have been deposited in the NCBI's database Gene Expression Omnibus (GEO) and are accessible through GEO series accession number GSE20547.

On a total of 54 oligonucleotide microarray chips representing over 39,000 transcripts each, we compared 17 PrPmtA and 15 PrPmtB with 22 WT RNA extracts. Separate comparisons were performed for striatum, midbrain/brainstem and cerebellum. Tissues from young versus old animals were compared for each brain region and mouse line. In view of the small fold changes expected from complex tissues such as brain, an extensive biomathematical workup including RMA-based normalization, fitting with a linear model, statistical ANOVA-based evaluation, stringent filtering, and representation of individual transcript changes per brain region in a decision matrix were applied to suppress background and to reveal the consistent effects. Overall, the gene expression pattern was very similar and only few alterations correlated to genotype or to progression with age (data not shown).

All data were first filtered to identify transcript regulations which correlated with the mutant genotype at both ages in all tissues. In this analysis, we found a significant effect of A53T-SNCA overexpression only on the transcript levels of *Homer1* and the less characterized *Slc16a12*, *C330006P03Rik* (downregulated*), Hspa1b*, *Myef2*, and *D14Ertd171e* (upregulated). HOMER1 proteins have an established role for glutamatergic corticostriatal neurons and in the morphogenesis of dendritic spines, assembling group I mGluRs (mGluR1 and mGluR5) and coupling them with inositol 1,4,5-trisphosphate (IP3) receptors and ion channels, while excessive synaptic activity or DRD1 agonists induce the dominant negative isoform HOMER1a as an immediate early gene (IEG) [22]. Alterations in the expression of *Homer1* in A53T-SNCA overexpressing mice from an early age in several brain areas may reflect altered activity and plasticity at excitatory synapses.

### Striatum-specific expression changes with age represent a progressively diminished DA response

The second biomathematical workup filtered expression changes with progression from young to old age occurring exclusively in the striatum of PrPmtA and PrPmtB mice, but not in the brainstem/midbrain or cerebellum, nor in WT tissues. Reflecting a positive control, the aging effect both in WT and mutant mice resulted consistently in all three brain regions in a significant upregulation of the astrocyte hypertrophy and neural atrophy marker glial fibrillary acidic protein (*Gfap*), together with immunity- and aging-relevant lysozyme transcripts (*Lzp-s* and *Lyzs*), and a significant downregulation of the imprinted, maternally expressed microRNA transcript gene trap locus 2 (*Gtl2*) (Table S2). In contrast, the transcript levels of 57 non-anonymous genes showed an expression change from young to old age selectively in the striatum of lines PrPmtA and PrPmtB, but not in the striatum of WT controls or in other brain regions (Table S2; references are included on various genes with established roles in DA neurotransmission, striatal synaptic plasticity and PD). Several of these transcripts are known to change expression in response to DA signaling, and invariably the changes observed were consistent with a diminished striatal DA response in PrPmtA and PrPmtB mice. Consistent reductions were observed in transcript levels of cannabinoid receptor 1 (*Cb_1_* or *Cnr1*, 0.39-fold), ephrin B2 (*Efnb2*, 0.49-fold), frequenin (*Freq*, 0.64-fold), Homer1 (*Homer1*, 0.5-fold) and phosphodiesterase 7B (*Pde7b*, 0.37-fold). Furthermore, a reduction was observed for the cyclic adenosine monophosphate (cAMP)-dependent transcription factor 2 (*Creb2* or *Atf2*, 0.44-fold), a gene known to be suppressed after axotomy of nigrostriatal neurons and in manifest PD. CB_1_, EFNB2 and HOMER1 have an established role in retrograde synaptic signaling, while ATF2 and PDE7B relate to cAMP second messenger levels downstream of DA receptors. In addition to HOMER1, CB_1_ is implicated in mGluR-induced, endocannabinoid-mediated synaptic plasticity of excitatory synapses of the striatum such as LTD [23]. Thus, the transcriptome data suggest that A53T-SNCA overexpression leads to a progressively diminished postsynaptic DA response and altered synaptic plasticity in the striatum.

### Independent techniques support the diminished transcriptional response to DA in striatum

**Figure 5:**
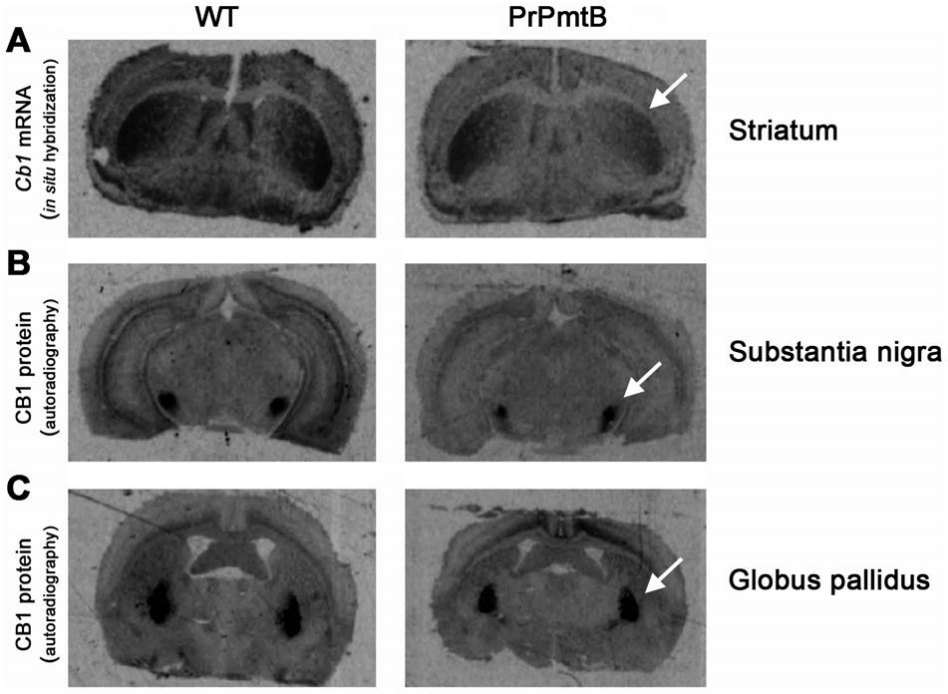
Decreased Cb_1_ expression on transcript and protein level. *In situ* hybridization and receptor autoradiography in A53T-SNCA overexpressing versus WT mice at old age show decreased levels of the cannabinoid receptor 1 (*Cb_1_*) mRNA in striatum (A; white arrow) and of CB_1_ protein in the substantia nigra (B; white arrow) as well as the globus pallidus (C; white arrow). Scale bar: 1 mm.

To extend this extensive transcriptome survey, we first employed radioactive *in situ* hybridization and receptor autoradiography to quantify the transcript and protein level of CB_1_ at its site of synthesis in the striatum and its sites of destination after anterograde axonal transport to the SN pars reticulata and the globus pallidus (GP). CB_1_ expression is known to be induced in response to chronic DA administration [24], [25]. Confirming the microarray data in the striatum of old A53T-SNCA overexpressing mice, the *Cb_1_* transcript level was significantly reduced by 15.4% (Fig. 5A, white arrow) in *in situ* hybridization experiments. Furthermore, receptor autoradiography experiments showed that CB_1_ binding levels in the SN and the GP were reduced by 43.3% (Fig. 5B, white arrow) and 20.2% (Fig. 5C, white arrow), respectively.

Second, we employed qPCR to analyze several striatal transcript levels in old A53T-SNCA overexpressing mice in comparison with corresponding WT and found significant downregulations of *Atf2* (PrPmtA: −19%, p = 0.0299; PrPmtB: −31%, p = 0.0014), *Cb_1_* (PrPmtA: −32%, p = 0.0471), *Homer1* (PrPmtA: −55%, p<0.0001; PrPmtB: −19%, p = 0.0348) and *Pde7b* (PrPmtB: −28%, p = 0.0344), genes known to be induced by DA signaling (Table S3). These results suggest that the old A53T-SNCA overexpressing mice exhibit a diminished DA response, despite cellular efforts to maximize DA signaling. We assume that the observed striatal transcriptome changes are secondary effects of compromised DA signaling through nigrostriatal A53T-SNCA overexpression since human SNCA immunoreactivity in the striatum of PrPmtA and PrPmtB mice is detectable only in synapses, but not in neurites or cell bodies [13].

Interestingly, also a parallel effort focusing on human PD midbrain to characterize the pathological expression profile of individual dopaminergic neurons confirmed dysregulation of *Freq* (Fig. S3). This independent evidence indicates that our mouse approach correctly identified a signaling molecule implicated in the human disease.

### Normal electrophysiological properties of striatal neurons in WT and A53T-SNCA overexpressing mice

**Figure 6:**
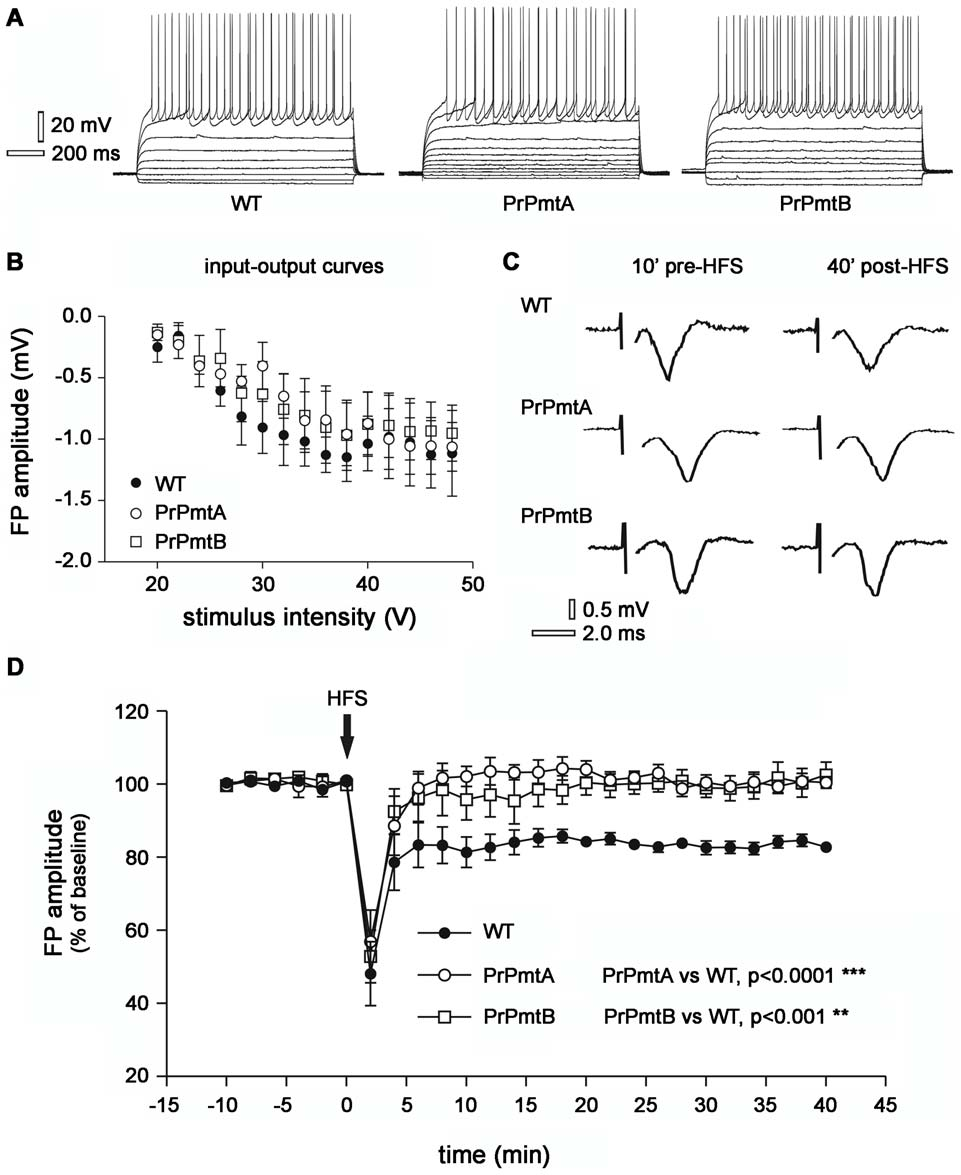
Electrophysiological analysis demonstrates absent long term depression (LTD) in corticostriatal slices of old mice. Voltage traces of striatal medium spiny neurons (MSNs) recorded in whole-cell patch-clamp during hyperpolarizing and depolarizing steps of current. Note the similar firing pattern discharge in the representative WT, PrPmtA and PrPmtB neuron (A). Graph showing a similar input-output curve of striatal field potential (FP) evoked by 200 µsec stimuli of increasing voltage intensities applied on corticostriatal fibers in WT, PrPmtA and PrPmtB mice (B). Representative examples of corticostriatal FP acquired 10 min before high frequency stimulation (HFS) protocol and 40 min after HFS in WT, PrPmtA and PrPmtB mice (C). Time-course of FP amplitudes showing the induction of LTD only in WT mice, whereas no long-lasting change of the FP amplitude in PrPmtA and PrPmtB mice is monitored up to 40 min following the HFS protocol application (D). The significance level was established at p<0.001 (**) and p<0.0001 (***).

Since the molecular data suggest altered dopaminergic modulation of striatal neurons and since dopaminergic synapses modulate the signaling at glutamatergic corticostriatal projections, we decided to perform a functional assessment of striatal postsynaptic responses. In order to first assess a functional role of A53T-SNCA in the regulation of intrinsic membrane properties, single cell recordings were obtained from corticostriatal slices prepared from WT and mice of both transgenic lines. Only neurons electrophysiologically identified as spiny neurons were considered for these experiments [26]. Whole-cell patch-clamp recordings showed that intrinsic membrane properties of striatal neurons were similar in the three groups tested and closely resembled the electrical activity described previously for rat striatal spiny neurons [27], [28], [29]. Indeed, the average resting membrane potential was −87±3 mV (n = 6) in WT, −87±4 mV (n = 4) in PrPmtA and −85±5 mV (n = 4) in PrPmtB mice. In all three groups neurons were silent at rest. The injection of positive current through the recording pipette induced a similar tonic firing discharge in all groups (Fig. 6A). Membrane rectification was present in the three groups. Striatal field potentials (FP) in WT, PrPmtA and PrPmtB mice were evoked every 10 sec by stimulating the cortical glutamatergic afferents to the striatum. Increasing stimulation levels (voltage: 20–48 V; duration: 200 µsec) of the corticostriatal pathway evoked FP of similar increasing amplitude in the three groups under investigation as shown in the plot of the input-out curves (Fig. 6B). Thus, the intrinsic excitability of striatal neurons in old A53T-SNCA overexpressing mice appears to be unchanged.

### Lack of induction of corticostriatal long term depression in A53T-SNCA overexpressing mice

Assessing the suggestion of altered synaptic plasticity in the transcriptome profile on a functional level, we studied LTD in corticostriatal slices from PrPmtA, PrPmtB and WT mice. A typical FP was recorded by an extracellular electrode following a single stimulation delivered from a stimulating electrode. At the beginning of each experiment, we measured the basal FP amplitude for 15–20 min. After the identification of a FP showing stable baseline amplitude, three trains of stimuli (100 Hz) were delivered. As previously described [26], this experimental protocol induced a LTD of FP amplitude which reached a steady state within 15 min from the tetanic stimulation (Fig. 6D, filled circle; FP reduction at 30 min: −18.6±1.9%, n = 15).

Conversely, while tetanic activation of cortical fibers showed significant LTD of the FP amplitude in WT animals, in PrPmtA and PrPmtB mice the FP did not show any long term change of synaptic efficacy (Fig. 6D). Figure 6C shows traces obtained from three single experiments recorded from slices prepared from representative PrPmtA, PrPmtB and WT mice. In the traces from the WT group the high frequency stimulation (HFS) induced a LTD (Fig. 6C, upper traces) while in the PrPmtA and PrPmtB groups LTD was completely absent (Fig. 6D, middle and bottom traces). LTD normally depends on the presence of physiological levels of DA and the D2 DA receptor activation is a permissive factor for LTD induction [30]. In order to valuate a possible effect of DA accumulation on corticostriatal LTD in PrPmtA and PrPmtB mice, we performed a set of experiments in the presence of the D2 DA receptor antagonist L-sulpiride (5 µM). However, also in these conditions HFS did not produce any long-term change of FP amplitude in PrPmtA and PrPmtB mice (n = 4, p>0.05, data not shown).

Thus, the electrophysiological data corroborate the conclusion from transcriptome studies and indicate a progressive impairment of synaptic plasticity in the striatum.

## Discussion

In view of a large body of controversial *in vitro* data on the physiological and pathological role of the presynaptic protein SNCA for dopaminergic signaling, this study characterized long term *in vivo* effects of SNCA mutations in the nigrostriatal dopaminergic projection of mice. We analyzed two independent transgenic mouse lines (PrPmtA and PrPmtB) using the neuron-specific prion protein (PrP) promoter to overexpress human A53T-SNCA. This missense mutation is responsible for the PARK1 variant of PD (3). An approximately 1.5-fold enhanced expression level mimics the SNCA gene duplication which causes the PARK4 variant of PD [4] and corresponds to the overexpression level observed in the nigrostriatal projection of these two transgenic mouse lines. Interestingly, despite the presence of two pathogenic SNCA mutations, these mice do not develop visible alpha-synuclein aggregation or detectable neuron loss during their two-year life span, but they display a progressive deficit in spontaneous locomotion, with unchanged coordination and strength [13]. These features mirror the characteristic movement impairment in PD which is due to deficient striatal DA signaling. In the current study we investigated the molecular mechanisms in the striatum which may underlie this movement deficit and neural dysfunction. Of course, A53T-SNCA overexpression would affect neural function also outside the nigrostriatal projection, but this model system is particularly well characterized and these mice were specifically generated and characterized with regard to the transgene presence in the substantia nigra and striatum.

Our observation of increased DA content in the striatum of old A53T-SNCA overexpressing mice was unexpected, but is similar to findings that the triple KO of the Parkinson genes PINK1, Parkin and DJ-1 results in an elevation of striatal DA in the absence of neurodegeneration [31]. Increased DA levels in the absence of neurodegeneration were also reported for PD mouse models deficient only in Parkin (PARK2) [32] and in some *in vitro* studies of A53T-SNCA [20], [33], [34]. In contrast, old SNCA overexpressing mice with visible protein aggregates and a loss of nerve terminals exhibit a reduction of striatal DA (15, 16). Our transgenic mice do not exhibit overt neurodegeneration, visible protein aggregation [13], a reduction in striatal TH (Fig. S1) or any genotype-dependent increase in the *Gfap* transcript (as a biomarker of astrocytosis, neurodegeneration and aging; Table S2). The progressively increased striatal DA levels observed in these animals (Fig. 2A and 2B) may thus be an early consequence of A53T-SNCA overexpression prior to overt neurodegeneration. Although it cannot be excluded that the observed increases in striatal DA reflect a toxic gain-of-function of the A53T-SNCA mutation, the observation of decreased striatal DA content in old SNCA-KO mice in this study (Fig. 2C and Fig. 2D) and also by an independent group [19] suggests a physiological modulation of DA content by the expression level of SNCA.

In the striata of A53T-SNCA overexpressing mice, DA signals in the extracellular space appear to be abnormal considering the upregulation of postsynaptic DA receptors and the transcriptional downregulation of extraneural COMT. Both events might constitute a compensatory effort to enhance dopaminergic signaling in response to pathologically low DA release or high DA reuptake. Indeed, also the DA accumulation might constitute a similar compensatory effort to enhance DA signaling. The notion of altered DA reuptake is conceivable in view of previous reports of protein association between SNCA and the dopamine transporter (DAT) [35]. On the other hand, a release problem also seems possible in the light of recent reports that deficiency in alpha- and gamma-synuclein leads to increased striatal DA release and a hyperdopaminergic phenotype [36]. Current evidence indicates that presynaptic SNCA acts together with the co-chaperone CSPα at the soluble NSF attachment protein receptor (SNARE) complex to regulate the exocytosis of neurotransmitter vesicles [1].

In spite of these progressive compensatory efforts, the analysis of striatal transcription suggests that the postsynaptic DA response is diminishing over time. The molecular profile of the striatal age-related changes was obtained through hypothesis-free, extensive transcriptome studies of striatal versus brainstem/midbrain and cerebellar tissue in young and old mice, and was validated by independent experiments utilizing qPCR, *in situ* hybridization and receptor autoradiography. Genotype analysis of all 54 microarray chips showed primarily *Homer1* changes which correlated with the A53T-SNCA genotype in brain regions beyond the striatum by as early as 6 months of age. HOMER1 modulates synaptogenesis through its role as a scaffold protein for mGluRs. In contrast to the constitutively expressed long splice variants HOMER1b/c, the short splice variant HOMER1a is induced by neurotransmission activity as an IEG, acts as endogenous negative modulator of the mGluR/IP_3_ receptor signaling complex and prevents excessive glutamate-induced neuronal activity [37]. The observation of genotype-dependent alterations of *Homer1* transcript levels thus suggests that A53T-SNCA overexpression affects glutamate neurotransmission throughout the brain and that *Homer1* expression is a useful marker of this effect. Within the striatum, the most striking changes were observed in several transcripts known to be regulated by dopaminergic innervation. DA signaling induces expression of the mGluR modulator HOMER1a [38], [39], CB_1_ [24], [25] and PDE7B [40]. Conversely, reduced expression levels of striatal *Homer1* and *mGluR1a* were previously observed after dopaminergic neuron death in the neurotoxic 1-methyl-4-phenyl-1,2,3,6-tetrahydropyridine (MPTP) model of PD [41], while animals with nigrostriatal denervation exhibit DA supersensitivity and abnormal expression of several IEGs, such as HOMER1a [9]. Axotomy of nigrostriatal dopaminergic neurons is reported to suppress the expression of *Atf2* [42], [43]. Therefore, the reduced transcript levels of all these genes in the striatum of old A53T-SNCA overexpressing mice indicate a progressively diminished postsynaptic DA response.

Our electrophysiological investigation was carried out since DA, CB_1_ and mGluRs are known prerequisites of LTD, the most prominent form of synaptic plasticity in the striatum, particularly for the indirect pathway MSNs. Striatal LTD requires the activation of DA receptors and mGluRs with the consequent release of endocannabinoids and a long-lasting inhibition of neurotransmitter release [23], [30], [44]. Neurotoxic DA depletion in animal models of end-stage PD is associated with a decrease in striatal synaptic plasticity, which progresses to a loss of spines and glutamatergic synapses on indirect pathway neurons [11]. The present analysis of genetic mouse models of PD demonstrates that loss of LTD is associated with the A53T-SNCA mutation or SNCA protein overexpression.

How can pathogenesis be induced, while visible Lewy-pathology is undetectable and insoluble alpha-synuclein is mainly monomeric in our mice? We speculate that increasing degrees of alpha-synuclein overexpression would first saturate all interaction sites within its physiological localizations such as the synapse and also the somatodendritic compartment, before solubility becomes impaired and then reversible as well as irreversible aggregation processes take place. In the synapse, an alpha-synuclein gain-of-physiological-function would interfere with the cycling of neurotransmitter vesicles [45] and could lead to an accumulation of dopamine, as was recently observed also in *C. elegans* [46].

**Figure 7:**
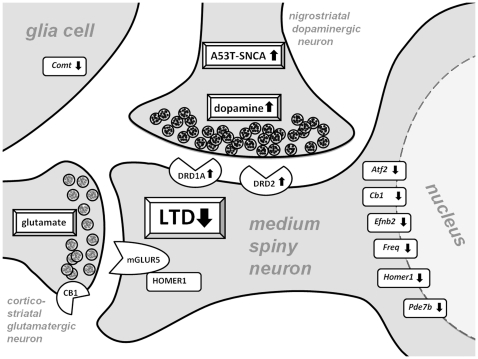
Synopsis of progressively abnormal synaptic signaling in old A53T-SNCA striata. This schematic illustration summarizes the progressive pathology of a striatal MSN dendritic spine. The MSN is innervated by nigrostriatal dopaminergic input where the presence of pathogenic overexpressed A53T-SNCA causes elevated presynaptic DA. In glia cells, the DA degradation enzyme COMT is transcriptionally downregulated, suggesting altered extracellular turnover of DA. Postsynaptically, the upregulation of DRD1A and DRD2 and the decreased transcript levels of several DA-inducible genes suggest a progressively diminishing striatal DA response. In parallel, the plasticity of corticostriatal glutamatergic synapses is affected through retrograde signaling, leading to the absence of LTD.

In conclusion, our findings (summarized in Fig. 7) demonstrate that A53T-SNCA overexpression in the murine nigrostriatal system is associated with a syndrome of progressive synaptic dysfunction, in the absence of neurodegeneration. This striatal dysfunction is evident by an enhancement of DA levels, a reduced expression of the extraneural DA degradation enzyme COMT and an upregulation of postsynaptic DA receptors, three abnormalities which may constitute compensatory efforts to maximize signal strength. Further, we identified Homer1 as molecular biomarker of this dysfunction and demonstrated a progressive impairment of synaptic plasticity at the molecular and electrophysiological level. Taken together, we suggest that these mice exhibit subtle changes in dopaminergic neurotransmission prior to the advent of neurodegeneration, and may thus be useful to understand molecular mechanisms occurring in early stages of the disease and to evaluate preventive therapies.

## Materials and Methods

### Animal welfare

Mice were housed at the FELASA-certified Central Animal Facility (ZFE) of Frankfurt Goethe University Medical School according to the animal husbandry guidelines of the German Animal Welfare Act, the Council Directive of November 24^th^, 1986 (86/609/EWG) with Annex II and the ETS123 (European Convention for the Protection of Vertebrate Animals).

All the experiments were conducted in conformity with the European Communities Council Directive of November 1986 (86/609/ECC).

### Animal breeding and dissection

Mice overexpressing A53T-SNCA and mice lacking endogenous SNCA were prepared as previously described [13], [47]. Animals were housed in the same room under 12/12 h light/dark cycle with food and water *ad libitum*. The two mouse lines overexpressing A53T-SNCA, PrPmtA and PrPmtB, were compared with their background strain FVB/N as WT controls while the SNCA-deficient (KO) mouse line was compared with its background strain 129sv/ev as WT control. Two age groups were analyzed for each genotype; 6-month-old animals represented adult animals prior to the development of motor impairment (young age), while at least 18-month-old animals exhibiting mild motor impairments represented the early stage of clinically manifest disease (old age). Following cervical dislocation, brainstem/midbrain, striatum and cerebellum were dissected, frozen in liquid nitrogen and stored at −80°C for molecular and biochemical analyses or fixed in 4% paraformaldehyde for immunohistochemistry. For *in situ* hybridization and autoradiography, brains were frozen in 2-methylbutane cooled in dry ice and then sliced in 20 µm-thick coronal sections, according to the Lehmann mouse brain atlas [48]. Sections were thaw-mounted onto RNase-free gelatin/chrome alum coated slides and dried briefly at 30°C and stored at −80°C until use. Adjacent sections were stained with cresyl-violet to verify the anatomical position.

### Immunoblots

Following tissue homogenization with radioimmunoprecipitation assay (RIPA) buffer (50 mM TRIS pH 8.0, 150 mM NaCl, 1% Triton X-100, 0.5% Na-deoxycholate, 0.1% SDS) supplemented with complete protease inhibitor cocktail (Roche, Mannheim, Germany), samples were incubated at 4°C, rotating for 20 min. Homogenates were centrifuged for 15 min at 4,500 *g* and 4°C and supernatants were collected. 20 µg of protein were separated in 10–12% tris-glycine polyacrylamide, depending on the size of the protein to be detected, and transferred onto a polyvinylidene fluoride (PVDF) membrane (Bio-Rad, Munich, Germany). Membranes were blocked with 5% fat-free milk in phosphate buffered saline/Tween (PBS/T; 0.1%), for 1 hour (h) at room temperature (RT) and incubated with the primary antibody [anti-SNCA, 1∶500 dilution (#610787, BD Transduction Laboratories, San Jose, CA, USA); anti-SNCA [4B12], 1∶200 dilution (#1904, abcam); anti-TH, 1∶1,000 dilution (#P40101, Pel-Freez, Rogers, AR, USA); anti-β-actin, 1∶15,000 dilution (#A5441, Sigma Aldrich, Hamburg, Germany)] in PBS/T overnight at 4°C, washed 3 times with PBS/T and incubated with the secondary antibody (alkaline phosphatase conjugated anti-mouse antibody, 1∶15,000 dilution (#NA931V, GE Healthcare, Munich, Germany) for 1 h at RT. After washing, the secondary antibody was detected with a chemiluminescent substrate (Pierce SuperSignal West Pico Chemiluminescent; Thermo Fisher Scientific Inc., Rockford, IL, USA). Densitometric analyses utilized TotalLab software (Amersham Biosciences, Freiburg, Germany).

### Sequential Extraction of **α**-Synuclein

Subcellular fractionation of human cerebral cortex samples and subdivided mice brain was performed as described previously [49]. Briefly, human tissue was homogenized in 10 vol and mouse tissue in 5 vol of TBS+ (Tris-buffered saline plus Complete protease inhibitor; Roche Diagnostics, Mannheim, Germany) and spun for 30 min at 120.000×g. The resulting supernatant represented the TBS+ soluble fraction. The pellet was then extracted sequentially with 1 ml of TBS+ containing 1% of Triton X-100, and TBS+, 1 M sucrose, following extraction with RIPA buffer (50 mM Tris-HCl, pH 7.4, 175 mM NaCl, 5 mM EDTA, 1% NP-40 and 0.5% sodium deoxycholate) and 0.1% SDS. The detergent-insoluble pellet was solubilized in 8 M urea/5% SDS. The supernatants were supplemented with glycerol and stored at −80°C. Protein extracts (37 µg each) were fractionated on 17% SDS-PAGE and blotted on a nitrocellulose membrane (Whatman, Dassel, Germany). Immunoblots were blocked in 5% dry milk in TBST buffer (10 mM Tris pH 7.5; 0.15 M NaCl; 0.1% Tween 20) and subsequently probed with 15G7-α-syn antibody (1∶20) (AG Scientific, San Diego, CA) and Mc42 (1∶3000) (Transduction laboratories, Lexington, KY). Anti-β-actin (1∶10.000) (A4700; Sigma) was used as loading control. Bound antibodies were visualized with horseradish peroxidase-conjugated secondary antibodies and enhanced chemiluminescence (ECL or ECL^plus^, GE Healthcare) and exposure to hyperfilm (GE Healthcare). Brain tissues subjected to sequential protein extraction were analyzed 3 times on different Western blots with similar results.

### HPLC assay of striatal DA and its metabolites

Method 1 (used in Würzburg): Striatal samples were thawed, weighed and sonicated in 0.05 M perchloric acid for 60 seconds [24] resulting in 10% (w/v) homogenate suspensions. These were centrifuged at 48,000 *g* for 20 min at 4°C, and 50 µl samples of the supernatants injected directly into a HPLC system with electrochemical detection (ED) (Gynkotek GmbH, Germering, Germany) for analysis of tissue DA according to the method as previously reported [50]. The HPLC system consisted of an AGILENT 1100 series (Bio-Rad, Munich, Germany), a Nucleosil 120-5C_18_ reverse phase (250×4.6 mm) analytical column (Macherey-Nagel, Düren, Germany), an electrochemical detector (model 1640; Bio-Rad, Munich, Germany), and a mobile phase (flow rate: 1.5 ml/min) containing 0.1 M sodium phosphate buffer, pH 3.9, 84% (v/v) and 16% (v/v) methanol containing 0.1 mM ethylenediaminetetraacetic acid (EDTA), 0.65 mM 1-octanesulfonic acid and 0.5 mM triethylamine. The detector potential was set at +0.75 V relative to the Ag/AgCl reference electrode. The signal from the detector was recorded and data analyses were performed using an AGILENT Chem Station for LC9D (Bio-Rad, Munich, Germany). Concentrations were calculated from the peak height with the aid of an external standard. The investigator was blind to the animal's genotype.

Method 2 (used in Madrid): Striata were homogenized in 20–40 volumes of cold 150 mM potassium phosphate buffer, pH 6.8. Each homogenate was aliquoted to allow parallel measurements of protein concentrations according to the method of Lowry as well as DA and homovanillic acid (HVA) content using HPLC-ED, following a previously published method [51]: One aliquot was diluted (1∶2) with 0.4 N perchloric acid containing 0.4 mM sodium disulfite, 0.9 mM EDTA and the corresponding internal standard (dihydroxybenzylamine for catecholamines). Samples were centrifuged for 3 min at 15,000 *g* and supernatants were injected directly into the HPLC system which consisted of the following components: The pump was an isocratic Spectra-Physics 8810. The column was a RP-18 (Spherisorb ODS-2; 150 mm, 4.6 mm, 5 µm particle size; Waters, Massachusetts, USA). The mobile phase, previously filtered and degassed, consisted of 100 mM citric acid, 100 mM sodium acetate, 1.2 mM heptane sulphonate, 1 mM EDTA and 7% methanol (pH 3.9). The flow rate was 0.8 ml/min. The effluent was monitored with a coulochemical detector (Coulochem II, ESA) using a procedure of oxidation/reduction (conditioning cell: +360 mV; analytical cell #1: +50 mV; analytical cell #2: −340 mV) that reaches a sensitivity of 50 nA (10 pg per sample). The signal was recorded on a Spectra-Physics 4290 integrator. The results were obtained from the peaks and calculated by comparison with the area under the corresponding internal standard peak. Values were expressed as ng per mg of protein.

### DRD1-/DRD2-Immunoblots

In contrast to other immunoblot experiments, the protein samples used for the detection of dopamine receptors DRD1 and DRD2 were not boiled at 95°C for 5 min before being loaded onto the gel after personal communication with the group that generated the DRD1A antibody. Instead, the protein samples were heated at 40°C for 5 min. The following mouse monoclonal antibodies were used: anti-DRD1A, 1∶200 dilution (#sc-33660, D1DR (SG2-D1a); Santa Cruz Biotechnology, Santa Cruz, CA, USA), anti-DRD2 1∶200 dilution (#sc-5303, D2DR (B-10); Santa Cruz Biotechnology, Santa Cruz, CA, USA), anti-glyceraldehyde-3-phosphate dehydrogenase (GAPDH), 1∶10,000 dilution (#CB1001, mAb (6C5); Calbiochem/Merck, Darmstadt, Germany) and anti-β-actin, 1∶10,000 dilution (#A5441, Clone AC-15; Sigma Aldrich, Hamburg, Germany).

### DRD1-/DRD2-Autoradiography

#### Preparation of brain slices

After cervical dislocation of the mice, the brains were carefully dissected, immediately frozen in dry ice cooled 2-methylbutane and stored at −80°C until use. Before the preparation of the 14 µm-thick horizontal brain slices, the brains were equilibrated in the cryostat chamber to a temperature of −15±1°C. Sections were melted onto super frosted glass slides (Menzel GmbH, Braunschweig, Germany), air dried and finally stored at −80°C until use.

#### Autoradiography of DRD1 and DRD2 binding

During DRD1 autoradiography, horizontal brain slices were incubated for 1 h at RT in a buffer containing 1 M Tris, 120 mM NaCl, 5 mM KCl, 2 mM CaCl_2_, 1 mM MgCl_2_ and 2 nM tritium [^3^H]-labeled SCH23390 (85 Ci/mmol). In case of DRD2 autoradiography, horizontal brain sections were incubated for 1 h on ice in a buffer containing 50 mM Tris citrate (pH 7.4), 120 mM NaCl and 0.5 nM [^3^H]-spiperone (15 Ci/mmol). To determine the non-specific binding of [^3^H]-SCH23390 as well as [^3^H]-spiperone, slices were incubated in the same way as previously described, both radioligands being replaced by high dosed DA (50 µM) and haloperidol (10 µM), respectively. The corresponding signal values were subtracted as non-specific background from the DRD1- and DRD2-specific signal values measured. Following incubation, slices were washed twice for 5 min in ice-cooled buffer, dipped in ice-cooled distilled water and finally dried under a stream of cold air. Autoradiographies were generated by exposing the slices to a [^3^H]-sensitive Fujifilm imaging plate for 4–7 days and scanning the signals via a Fujifilm FLA-7000 scanner. Measurement and analysis of the autoradiographies were performed by using Multigauge FujiFilm software.

#### Data Analysis

Digital photo-stimulated luminescence values per area (PSL/mm^2^) for both hemispheres of each horizontal brain slice were evaluated. Only those hemispheres were included for analysis, which differed less than 20%. Mean values of the two hemispheres of each slice were calculated and the corresponding background values were subtracted, respectively. The data of each individual experiment was checked for biological outliers.

Finally, the data of three independent DRD1 autoradiography experiments as well as the data of two independent DRD2 autoradiography experiments was normalized to the WT signals on the corresponding films and taken together. Student's t-test was used to determine significance.

### Transcriptome analyses with oligonucleotide microarray chips

Tissue was dissected from the brain of young mice (2 WT/2 PrPmtA/2 PrPmtB striata, 2 WT/2 PrPmtA/2 PrPmtB brainstems/midbrains, 2 WT/2 PrPmtA/2 PrPmtB cerebella) and old mice (4 WT/2 PrPmtA/2 PrPmtB striata, 6 WT/4 PrPmtA/3 PrPmtB brainstems/midbrains, 6 WT/5 PrPmtA/4 PrPmtB cerebella). Tissues from individual, particularly old mice up to 28 months age were included here to strengthen the definition of progression markers reflecting old age. Double-stranded cDNA was synthesized from total RNA extracts from the brain tissues using a Superscript Choice Kit (Invitrogen) with a T7-d(T)_24_ primer incorporating a T7 RNA polymerase promoter (Metabion, Martinsried, Germany). Subsequently, cRNA was prepared and biotin labeled by *in vitro* transcription (Enzo Life Sciences, Lörrach, Germany). Labeled cRNA was fragmented by incubation for 35 min at 94°C min in the presence of 40 mM Tris-OAc (pH 8.1), 100 mM KOAc, and 30 mM MgOAc. Labeled, fragmented cRNA (15 µg) was hybridized for 16 h at 45°C to a 430 2.0 mouse genome array (Affymetrix, Santa Clara, CA, USA). After hybridization, gene chips were automatically washed and stained with streptavidin–phycoerythrin using a fluidics station. The probe arrays were scanned at 0.7 µm resolution using a Genechip System Scanner 3000. Scanned images were subjected to visual inspection and analyzed using the Affymetrix's GCOS version 1.3.

The biomathematical analyses were performed by the IMGM Institute (Martinsried, Germany). To define the influence of the factors genotype or age on the transcriptome, linear models were applied. To determine the effect of genotype, the overlap between PrPmtA versus WT, and PrPmtB versus WT, changes were examined. For the mathematical-statistical assessment of data, their visualization and functional correlation, the software platform R (version 2.4.0) and the pertinent bioconductor packages Limma and Affy as well as the Spotfire Decision Site for Functional Genomics 9.0 and diverse tools at the panther website (http://www.pantherdb.org) were used. Initially, the expression data from all chips were normalized with the RMA (Robust Multichip Average) method to yield log_2_-transformed signal values. Global gene expression was compared between chips using scatter plots and Pearson's R correlation coefficients. The signal values were then averaged for the individual subgroups and transformed to linear scales to derive fold change values. Differences between subgroups were extracted as contrasts and analyzed with the moderated F-test (empirical Bayes method) including a correction step for multiple testing with the 5%-FDR-based method of Benjamini and Hochberg. To attribute significant regulations to individual genes, a decision matrix was generated based on the function decide tests within the Limma option nestedF, where significant up- or downregulations are represented by values of +1 or −1, respectively. All microarray data discussed in this publication are MIAME compliant; raw data have been deposited in the MIAME compliant NCBI's database Gene Expression Omnibus (GEO) [49] and are accessible through GEO series accession number GSE20547.

### *In situ* hybridization of Cb_1_ mRNA

Analyses of *Cb_1_* transcript levels were performed as previously described [52]. Briefly, sections were fixed in 4% paraformaldehyde for 5 min, rinsed twice in PBS, acetylated by incubation in 0.25% acetic anhydride and prepared in 0.1 M triethanolamine/0.15 M sodium chloride (pH 8.0) for 10 min. They were rinsed again in 0.3 M sodium chloride/0.03 M sodium citrate, pH 7.0, dehydrated and delipidated via an ethanol/chloroform series. A mixture (1∶1∶1) of the three 48-mer oligonucleotide probes complementary to bases 4–51, 349–396 and 952–999 of the rat *Cb_1_* cDNA (Du Pont NEN; the specificity of the probes used was assessed by Northern blot analysis) was 3′-end labeled with [^35^S]-dATP using terminal deoxynucleotidyl transferase. The sections were hybridized with [^35^S]-labeled oligonucleotide probes (7.5×10^5^ dpm per section), washed, exposed to X-ray film (Kodak) for one week and developed (D-19, Kodak) for 6 min at 20°C. The intensity of the hybridization signal was assessed by measuring the grey levels in the autoradiographic films using computer-assisted videodensitometry. Adjacent brain sections were co-hybridized with a 100-fold excess of cold probe or with RNase to determine the specificity of the signal (data not shown). Details of this procedure have been published previously [53].

### Autoradiography of CB_1_ binding

The protocol used was previously described. Briefly, slide mounted brain sections were incubated for 2.5 h at 37°C in a buffer containing 50 mM TRIS with 5% bovine serum albumin (BSA, fatty acid-free), pH 7.4, and 10 nM [^3^H]-CP-55,940 (Du Pont NEN) prepared in the same buffer, in the absence or the presence of 10 µM non-labeled CP-55,940 (Sigma Aldrich, Hamburg, Germany) to determine the total and the non-specific binding, respectively. Following incubation, slides were washed in 50 mM TRIS buffer with 1% BSA (fatty acid-free), pH 7.4, for 4 h (2×2 h) at 0°C, dipped in ice-cold distilled water and dried under a stream of cool dry air. Autoradiograms were generated by apposing the labeled tissues, together with autoradiographic standards ([^3^H] micro-scales; Amersham Biosciences, Freiburg, Germany), to [^3^H]-sensitive film ([^3^H]-Hyperfilm; Amersham Biosciences, Freiburg, Germany) for a period of 2 weeks. Autoradiograms were developed (D-19, Kodak) for 4 min at 20°C, and the films were analyzed and quantified in a computer-assisted videodensitometer using standard curves generated from [^3^H]-standards.

### Transcript level assays with quantitative real-time RT-PCR

Total RNA was extracted from mouse brain tissues with TRIZOL (Invitrogen, Karlsruhe, Germany) and digested with DNase (amplification grade I; Invitrogen, Karlsruhe, Germany) following the manufacturers' instructions. In the case of the striata, RNeasy Lipid Tissue Mini Kit (Qiagen, Hilden, Germany) was used for RNA extraction. 2 µg of DNase treated RNA were reverse transcribed in a 36 µl reaction, using pd(N)_6_ and NotI-d(T)_18_ primers (First Strand cDNA Synthesis Kit; Amersham Biosciences, Freiburg, Germany). All qPCR reactions were carried out in 20 µl containing 0.5 µl of cDNA template, 10 µl of 2× TaqMan® Universal Master Mix (Applied Biosystems, Foster City, CA, USA) and 1 µl TaqMan® gene expression assay on an ABI Prism 5700 sequence detection system. The following PCR protocol was used: “stage 1” 2 min at 50°C; “stage 2” 10 min at 95°C; “stage 3” 15 sec at 95°C/40 sec at 60°C (40 repeats). The following mouse TaqMan® assays were employed: *SNCA* Hs01103383_m1, *Atf2* Mm00833804_g1, *Cb_1_* Mm00432621_s1, *Comt* Mm00514377_m1, *Dat* ( = *Slc6a3*) Mm00438388_m1, *Ddc* Mm00516688_m1, *Gch1* Mm00514993_m1, *Homer1* Mm00516275_m1, *Maoa* Mm00558004_m1, *Maob* Mm00555412_m1, *Pde7b* Mm00450009_m1, *Spr* Mm00488430_m1, *Th* Mm00447546_m1, *Vmat2* ( = *Slc18a2*) Mm00553058_m1 and *Tbp* Mm00446973_m1 as internal standard for normalization. Expression changes were analyzed with the 2^−ΔΔCt^ method [52].

Analysis of frequenin mRNA levels in individual neuromelanin-positive SN DA neurons from human *post mortem* Parkinson's disease and matched control brains via UV-lasermicrodissection (UV-LMD) and qPCR was carried out essentially as described [54] using the human TaqMan® assay *Freq* Hs00977274 (60 bp, spanning exon boundary 4-5; Applied Biosystems). Standard curve for qPCR quantification was generated using serial dilutions of cDNA (3, 0.3 and 0.03 ng) derived from human SN tissue RNA (Ambion) as template in duplicates in n = 3 independent qPCR runs. Fluorescence threshold for all data for Ct analysis was 0.3; human *freq* cDNA standard-curve parameters: slope: 3.24±0.01; R2: 1.00±0.01, Y-Intercept: 30.82±0.34.

### Preparation and maintenance of slices for electrophysiological recordings

Corticostriatal coronal slices (thickness, 270 nm) were cut from 18-month-old male WT, PrPmtA and PrPmtB mice (n = 10 mice per genotype) using a vibratome. Preparation and maintenance of rat corticostriatal slices have been previously described [26], [55], [56]. A single slice was transferred to a recording chamber and submerged in a continuously flowing Kreb's solution (34°C; 2.5–3 ml/min) bubbled with a 95% O_2_–5% CO_2_ gas mixture. The composition of the solution was: 126 mM NaCl, 2.5 mM KCl, 1.2 mM MgCl_2_, 1.2 mM NaH_2_PO_4_, 2.4 mM CaCl_2_, 10 mM glucose, and 25 mM NaHCO_3_.

### Electrophysiology

Striatal MSNs were visualized using differential interference contrast (Nomarski) and infrared microscopy (Olympus Europa GmbH, Hamburg, Germany). Patch-clamp recordings were performed with borosilicate glass pipettes (4–7 MΩ) filled with intracellular solution: 145 mM K^+^-gluconate, 0.1 mM CaCl_2_, 2 mM MgCl_2_, 0.1 mM ethyleneglycoltetraacetic acid (EGTA), 10 mM 4-(2-hydroxyethyl)-1-piperazineethanesulfonic acid (HEPES), 0.3 mM guanosine triphosphate (GTP) and 2 mM Mg-ATP, adjusted to pH 7.3 with KOH. Signals were amplified with a Multiclamp 700B amplifier, recorded in current-clamp mode and stored on PC using pClamp10 (Axon Instruments, Foster City, CA, USA). Whole-cell access resistance was 5–30 MΩ. For extracellular recordings an Axoclamp 2B amplifier (Axon Instruments, Foster City, CA, USA) was used and recording electrodes, filled with 2 mol/l NaCl (15–20 MΩ), were invariably placed within the striatum. The FP amplitude was defined as the average of the amplitude from the peak of the early positivity to the peak negativity and the amplitude from the peak negativity to peak late positivity [57]. A corticostriatal FP was evoked every 10 sec by means of a bipolar electrode connected to a stimulator unit (Grass Telefactor, USA). The stimulating electrode was located in the white matter between the cortex and the striatum to activate corticostriatal fibers. For the input-output curve, a stimulus of increasing voltage intensity and constant duration (200 µsec) was delivered every 10 sec prior the onset of each experiment. Quantitative data are expressed as a percentage of the FP amplitudes in respect to the relative control amplitude values, the latter representing the mean of responses recorded during a stable period. For induction of LTD, a conditioning high frequency stimulation (HFS) protocol of three trains (3 sec duration at 20 sec intervals) was delivered at 100 Hz frequency. Only one experiment involving a conditioning HFS protocol was conducted on a single slice. Offline analysis and statistic was performed using Clampfit 10 (Axon Instruments, Foster City, CA, USA) and GraphPad Prism 3.02 (GraphPad Software, San Diego CA, USA) software. Analysis of variance (ANOVA) and Bonferroni's *post hoc* test were used for statistical analysis. Values given in the figures and text are mean ± standard error of the mean (SEM).

### Statistical analysis

Statistical analysis of DA concentration, *in situ* hybridization, receptor autoradiography and qPCR was performed using unpaired t-tests via the Prism 3 software (GraphPad, La Jolla, CA, USA). Data are presented as mean ± SEM. Significant differences were highlighted with asterisks (* p<0.05; ** p<0.01; *** p<0.001).

## Supporting Information

Figure S1 SNCA/A53T-SNCA and TH immunoblots of striatal protein extracts at young and old age. Illustration of tyrosine hydroxylase (TH; left panel) and α-synuclein (murine SNCA and human A53T-SNCA; right panel) protein levels in all mutant mouse lines (PrPmtA, PrPmtB and KO) as well as wild-type (WT) animals (FVB/N and 129sv/ev) at both ages investigated. Both TH and SNCA/A53T-SNCA immunoblots demonstrate that dopaminergic innervation to the striatum is preserved at both ages studied and that SNCA does not vary with age in these animals. As expected, no endogenous SNCA was present in striatal tissues of the KO mice (data not shown). Equal loading was controlled with β-actin. N = 4 animals per age and genotype.
(5.91 MB TIF)

Figure S2 Insoluble alpha-synuclein in human PD and old A53T-SNCA brain. Tissue samples were subjected to sequential protein extraction as described in materials and methods, including cortex tissue (A) from a patient with Parkinson's disease (PD; BrainNet, Munich) as positive control and (B) from healthy human individual as negative control, together with samples of striatum and midbrain from (B) 18-month-old PrPmtA mice and (C) PrPmtB mice. The immunoblots were detected with an antibody recognizing the NAC fragment of human and mouse alpha-synuclein. Thus, signals in TBS-soluble fractions correspond to soluble alpha-synuclein, T/T signals correspond to membrane-bound alpha-synuclein and bands in the urea (U) fractions represents insoluble alpha-synuclein. (A) In the analyzed disease patient, alpha-synuclein was detected in the soluble (TBS), membrane-bound (T/T) and in the insoluble (U) extracts. (B) Comparable to signals seen in the PD patient, also the the insoluble fraction of PrPmtA nigrostriatal tissue contained monomeric alpha-synuclein and faint high molecular bands. In a healthy human control individual, alpha-synuclein signal was detected only in soluble and membrane-bound fraction, but not in the insoluble fraction of brain tissue. (C) A prominent insoluble monomeric (α-Syn)1 signal was detectable in midbrain of a 18-month-old PrPmtB transgenic mice (left panel), and (right panel) insoluble alpha-synuclein was found as high molecular forms (α -Syn)n in striatum and as monomeric species in striatum and midbrain of a second 18-month-old PrPmtB mouse, but not in a third analyzed mouse. Bands of insoluble anti-alpha-synuclein-immunoreactivity were highlighted with asterisks. Beta-actin was used as loading control.
(4.54 MB TIF)

Figure S3 Altered frequenin mRNA expression of individual substantia nigra dopaminergic neuron from human PD post mortem midbrain via UV-LMD and qPCR. Neuromelanin-positive [NM(+)] neurons were isolated via UV-laser microdissection from cresylviolet-stained horizontal midbrain cryo-sections from PD and control brains (n = 5 each, provided from the German BrainNet). Left: Scatter plot of frequenin mRNA levels in PD and control brains. mRNA expression of each pool of 15 NM- and tyrosine hydroxylase- (TH-) positive SN DA neurons is given as pg-equivalents of total cDNA, derived from human SN tissue, per cell (determined via qPCR, standard curve quantification). Bars represent mean±SEM frequenin mRNA levels for all analyzed SN DA pools of each individual brain, (age, gender and RNA integrity number (RIN) for each brain is given at the bottom). Right: Mean mRNA level of frequenin was significantly higher in individual NM(+) SN DA neurons from PD brains compared to those of controls (PD: 17.89±1.54 pg, n = 33; controls 6.78±1.07, n = 27; p = 2×10∧−7).
(5.21 MB TIF)

Table S1 Expression analysis of candidate genes involved in dopamine homeostasis via qPCR. Transcript expression levels of enzymes involved in dopamine synthesis or degradation as well as transporters responsible for reuptake and sequestration of dopamine in presynaptic storage vesicles in the striatum of old A53T-SNCA overexpressing mice (PrPmtA: n = 3–6; PrPmtB: n = 3–8) and corresponding wild-type animals (WT: n = 3–6) were studied via qPCR. The only transcript, which was consistently altered in both transgenic mouse lines PrPmtA and PrPmtB at old age, was the transcript for the extraneuronal dopamine-degrading enzyme catechol-ortho-methyltransferase (Comt). Significant transcript expression changes are presented in percentage ± SEM and highlighted with black arrows and asterisks (** p<0.01; *** p<0.001).
(0.03 MB XLS)

Table S2 Transcriptome excerpt of striatum, brainstem/midbrain and cerebellum of young and old A53T-SNCA overexpressing mice. Significantly down- (-1) or up- (+1) regulated transcripts are clustered for DA-induced genes, alpha-synuclein interacting proteins, signaling proteins, transcription/translation and adhesion proteins. References with relevance for PD pathogenesis are given for each transcript.
(0.04 MB XLS)

Table S3 Independent validation of transcriptional dysregulation of dopamine-induced genes by qPCR. Striatal mRNA expression levels in old PrPmtA and PrPmtB mouse brains were documented. Significant changes are highlighted with arrows, the significance values is illustrated by asterisks (* p<0.05; ** p<0.01; *** p<0.001). N = 4 per genotype.
(0.03 MB XLS)

## References

[1] Chandra S, Gallardo G, Fernandez-Chacon R, Schluter OM, Sudhof TC (2005). Alpha-synuclein cooperates with CSPalpha in preventing neurodegeneration.. Cell.

[2] Liu S, Ninan I, Antonova I, Battaglia F, Trinchese F (2004). alpha-Synuclein produces a long-lasting increase in neurotransmitter release.. Embo J.

[3] Polymeropoulos MH, Lavedan C, Leroy E, Ide SE, Dehejia A (1997). Mutation in the alpha-synuclein gene identified in families with Parkinson's disease.. Science.

[4] Singleton AB, Farrer M, Johnson J, Singleton A, Hague S (2003). alpha-Synuclein locus triplication causes Parkinson's disease.. Science.

[5] Spillantini MG, Crowther RA, Jakes R, Hasegawa M, Goedert M (1998). alpha-Synuclein in filamentous inclusions of Lewy bodies from Parkinson's disease and dementia with lewy bodies.. Proc Natl Acad Sci U S A.

[6] Braak H, Del Tredici K, Rub U, de Vos RA, Jansen Steur EN (2003). Staging of brain pathology related to sporadic Parkinson's disease.. Neurobiol Aging.

[7] Krasnova IN, Betts ES, Dada A, Jefferson A, Ladenheim B (2007). Neonatal dopamine depletion induces changes in morphogenesis and gene expression in the developing cortex.. Neurotox Res.

[8] Seeman P, Niznik HB (1990). Dopamine receptors and transporters in Parkinson's disease and schizophrenia.. Faseb J.

[9] Gerfen CR (2000). Molecular effects of dopamine on striatal-projection pathways.. Trends Neurosci.

[10] Calabresi P, Mercuri NB, Di Filippo M (2009). Synaptic plasticity, dopamine and Parkinson's disease: one step ahead.. Brain.

[11] Day M, Wang Z, Ding J, An X, Ingham CA (2006). Selective elimination of glutamatergic synapses on striatopallidal neurons in Parkinson disease models.. Nat Neurosci.

[12] Cenci MA, Lundblad M (2006). Post- versus presynaptic plasticity in L-DOPA-induced dyskinesia.. J Neurochem.

[13] Gispert S, Del Turco D, Garrett L, Chen A, Bernard DJ (2003). Transgenic mice expressing mutant A53T human alpha-synuclein show neuronal dysfunction in the absence of aggregate formation.. Mol Cell Neurosci.

[14] Masliah E, Rockenstein E, Veinbergs I, Mallory M, Hashimoto M (2000). Dopaminergic loss and inclusion body formation in alpha-synuclein mice: implications for neurodegenerative disorders.. Science.

[15] Richfield EK, Thiruchelvam MJ, Cory-Slechta DA, Wuertzer C, Gainetdinov RR (2002). Behavioral and neurochemical effects of wild-type and mutated human alpha-synuclein in transgenic mice.. Exp Neurol.

[16] Tofaris GK, Garcia Reitbock P, Humby T, Lambourne SL, O'Connell M (2006). Pathological changes in dopaminergic nerve cells of the substantia nigra and olfactory bulb in mice transgenic for truncated human alpha-synuclein(1-120): implications for Lewy body disorders.. J Neurosci.

[17] Abeliovich A, Schmitz Y, Farinas I, Choi-Lundberg D, Ho WH (2000). Mice lacking alpha-synuclein display functional deficits in the nigrostriatal dopamine system.. Neuron.

[18] Chandra S, Fornai F, Kwon HB, Yazdani U, Atasoy D (2004). Double-knockout mice for alpha- and beta-synucleins: effect on synaptic functions.. Proc Natl Acad Sci U S A.

[19] Al-Wandi A, Ninkina N, Millership S, Williamson SJ, Jones PA (2008). Absence of alpha-synuclein affects dopamine metabolism and synaptic markers in the striatum of aging mice.. Neurobiol Aging.

[20] Lotharius J, Barg S, Wiekop P, Lundberg C, Raymon HK (2002). Effect of mutant alpha-synuclein on dopamine homeostasis in a new human mesencephalic cell line.. J Biol Chem.

[21] Sidhu A, Wersinger C, Vernier P (2004). alpha-Synuclein regulation of the dopaminergic transporter: a possible role in the pathogenesis of Parkinson's disease.. FEBS Lett.

[22] Thomas U (2002). Modulation of synaptic signalling complexes by Homer proteins.. J Neurochem.

[23] Kreitzer AC, Malenka RC (2007). Endocannabinoid-mediated rescue of striatal LTD and motor deficits in Parkinson's disease models.. Nature.

[24] Giuffrida A, Parsons LH, Kerr TM, Rodriguez de Fonseca F, Navarro M (1999). Dopamine activation of endogenous cannabinoid signaling in dorsal striatum.. Nat Neurosci.

[25] Zeng BY, Dass B, Owen A, Rose S, Cannizzaro C (1999). Chronic L-DOPA treatment increases striatal cannabinoid CB1 receptor mRNA expression in 6-hydroxydopamine-lesioned rats.. Neurosci Lett.

[26] Calabresi P, Ascone CM, Centonze D, Pisani A, Sancesario G (1997). Opposite membrane potential changes induced by glucose deprivation in striatal spiny neurons and in large aspiny interneurons.. J Neurosci.

[27] Calabresi P, Mercuri NB, Bernardi G (1990). Synaptic and intrinsic control of membrane excitability of neostriatal neurons. II. An in vitro analysis.. J Neurophysiol.

[28] Jiang ZG, North RA (1991). Membrane properties and synaptic responses of rat striatal neurones in vitro.. J Physiol.

[29] Kita H, Kitai ST (1994). The morphology of globus pallidus projection neurons in the rat: an intracellular staining study.. Brain Res.

[30] Calabresi P, Picconi B, Tozzi A, Di Filippo M (2007). Dopamine-mediated regulation of corticostriatal synaptic plasticity.. Trends Neurosci.

[31] Kitada T, Tong Y, Gautier CA, Shen J (2009). Absence of nigral degeneration in aged parkin/DJ-1/PINK1 triple knockout mice.. J Neurochem.

[32] Itier JM, Ibanez P, Mena MA, Abbas N, Cohen-Salmon C (2003). Parkin gene inactivation alters behaviour and dopamine neurotransmission in the mouse.. Hum Mol Genet.

[33] Lotharius J, Brundin P (2002). Impaired dopamine storage resulting from alpha-synuclein mutations may contribute to the pathogenesis of Parkinson's disease.. Hum Mol Genet.

[34] Mosharov EV, Staal RG, Bove J, Prou D, Hananiya A (2006). Alpha-synuclein overexpression increases cytosolic catecholamine concentration.. J Neurosci.

[35] Wersinger C, Sidhu A (2005). Disruption of the interaction of alpha-synuclein with microtubules enhances cell surface recruitment of the dopamine transporter.. Biochemistry.

[36] Senior SL, Ninkina N, Deacon R, Bannerman D, Buchman VL (2008). Increased striatal dopamine release and hyperdopaminergic-like behaviour in mice lacking both alpha-synuclein and gamma-synuclein.. Eur J Neurosci.

[37] Tappe A, Kuner R (2006). Regulation of motor performance and striatal function by synaptic scaffolding proteins of the Homer1 family.. Proc Natl Acad Sci U S A.

[38] Henning J, Koczan D, Glass A, Karopka T, Pahnke J (2007). Deep brain stimulation in a rat model modulates TH, CaMKIIa and Homer1 gene expression.. Eur J Neurosci.

[39] Yamada H, Kuroki T, Nakahara T, Hashimoto K, Tsutsumi T (2007). The dopamine D1 receptor agonist, but not the D2 receptor agonist, induces gene expression of Homer 1a in rat striatum and nucleus accumbens.. Brain Res.

[40] Sasaki T, Kotera J, Omori K (2004). Transcriptional activation of phosphodiesterase 7B1 by dopamine D1 receptor stimulation through the cyclic AMP/cyclic AMP-dependent protein kinase/cyclic AMP-response element binding protein pathway in primary striatal neurons.. J Neurochem.

[41] Kuwajima M, Dehoff MH, Furuichi T, Worley PF, Hall RA (2007). Localization and expression of group I metabotropic glutamate receptors in the mouse striatum, globus pallidus, and subthalamic nucleus: regulatory effects of MPTP treatment and constitutive Homer deletion.. J Neurosci.

[42] Pearson AG, Curtis MA, Waldvogel HJ, Faull RL, Dragunow M (2005). Activating transcription factor 2 expression in the adult human brain: association with both neurodegeneration and neurogenesis.. Neuroscience.

[43] Winter C, Schenkel J, Burger E, Eickmeier C, Zimmermann M (2000). The immunophilin ligand FK506, but not GPI-1046, protects against neuronal death and inhibits c-Jun expression in the substantia nigra pars compacta following transection of the rat medial forebrain bundle.. Neuroscience.

[44] Calabresi P, Centonze D, Bernardi G (2000). Electrophysiology of dopamine in normal and denervated striatal neurons.. Trends Neurosci.

[45] Nemani VM, Lu W, Berge V, Nakamura K, Onoa B (2010). Increased expression of alpha-synuclein reduces neurotransmitter release by inhibiting synaptic vesicle reclustering after endocytosis.. Neuron.

[46] Cao P, Yuan Y, Pehek EA, Moise AR, Huang Y Alpha-synuclein disrupted dopamine homeostasis leads to dopaminergic neuron degeneration in Caenorhabditis elegans.. PLoS One.

[47] Cabin DE, Shimazu K, Murphy D, Cole NB, Gottschalk W (2002). Synaptic vesicle depletion correlates with attenuated synaptic responses to prolonged repetitive stimulation in mice lacking alpha-synuclein.. J Neurosci.

[48] Lehmann A (1974). Atlas stereotaxique du Cerveau de la Souris Centre National de la Recherche Scientifique, Paris.

[49] Seidel K, Schols L, Nuber S, Petrasch-Parwez E, Gierga K First appraisal of brain pathology owing to A30P mutant alpha-synuclein.. Ann Neurol.

[50] Romero J, de Miguel R, Garcia-Palomero E, Fernandez-Ruiz JJ, Ramos JA (1995). Time-course of the effects of anandamide, the putative endogenous cannabinoid receptor ligand, on extrapyramidal function.. Brain Res.

[51] Rubino T, Massi P, Patrini G, Venier I, Giagnoni G (1994). Chronic CP-55,940 alters cannabinoid receptor mRNA in the rat brain: an in situ hybridization study.. Neuroreport.

[52] Lastres-Becker I, Berrendero F, Lucas JJ, Martin-Aparicio E, Yamamoto A (2002). Loss of mRNA levels, binding and activation of GTP-binding proteins for cannabinoid CB1 receptors in the basal ganglia of a transgenic model of Huntington's disease.. Brain Res.

[53] Herkenham M, Lynn AB, Johnson MR, Melvin LS, de Costa BR (1991). Characterization and localization of cannabinoid receptors in rat brain: a quantitative in vitro autoradiographic study.. J Neurosci.

[54] Grundemann J, Schlaudraff F, Haeckel O, Liss B (2008). Elevated alpha-synuclein mRNA levels in individual UV-laser-microdissected dopaminergic substantia nigra neurons in idiopathic Parkinson's disease.. Nucleic Acids Res.

[55] Calabresi P, Gubellini P, Picconi B, Centonze D, Pisani A (2001). Inhibition of mitochondrial complex II induces a long-term potentiation of NMDA-mediated synaptic excitation in the striatum requiring endogenous dopamine.. J Neurosci.

[56] Picconi B, Centonze D, Hakansson K, Bernardi G, Greengard P (2003). Loss of bidirectional striatal synaptic plasticity in L-DOPA-induced dyskinesia.. Nat Neurosci.

[57] Costa C, Belcastro V, Tozzi A, Di Filippo M, Tantucci M (2008). Electrophysiology and pharmacology of striatal neuronal dysfunction induced by mitochondrial complex I inhibition.. J Neurosci.

