# Supplementary figures and images for: A53T-Alpha-Synuclein Overexpression Impairs Dopamine Signaling and Striatal Synaptic Plasticity in Old Mice
Source: PLoS One. 2010 Jul 7;5(7):e11464. doi: 10.1371/journal.pone.0011464 (PMC2898885; doi:10.1371/journal.pone.0011464)

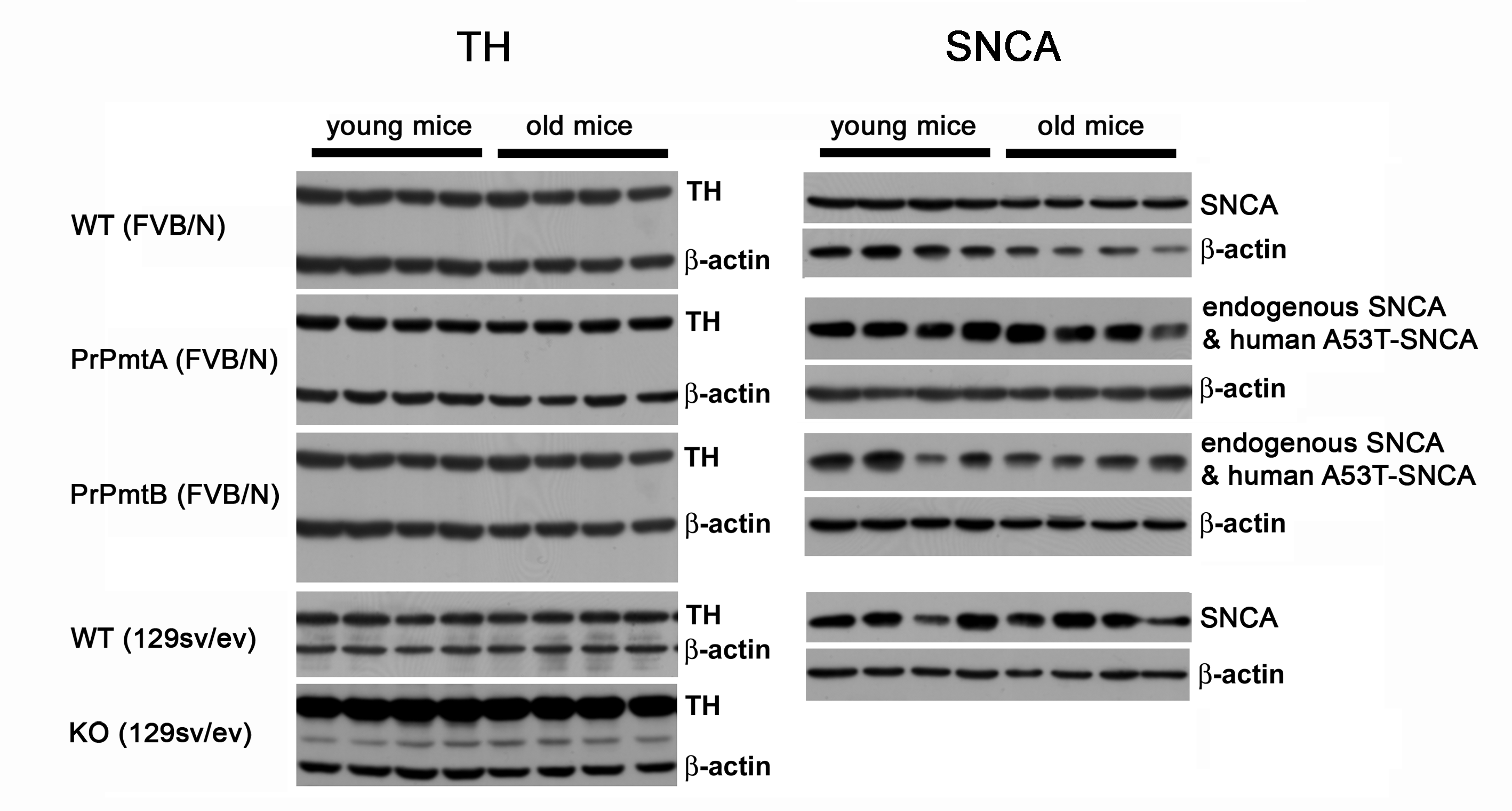

Supplement: Figure S1 — SNCA/A53T-SNCA and TH immunoblots of striatal protein extracts at young and old age. Illustration of tyrosine hydroxylase (TH; left panel) and α-synuclein (murine SNCA and human A53T-SNCA; right panel) protein levels in all mutant mouse lines (PrPmtA, PrPmtB and KO) as well as wild-type (WT) animals (FVB/N and 129sv/ev) at both ages investigated. Both TH and SNCA/A53T-SNCA immunoblots demonstrate that dopaminergic innervation to the striatum is preserved at both ages studied and that SNCA does not vary with age in these animals. As expected, no endogenous SNCA was present in striatal tissues of the KO mice (data not shown). Equal loading was controlled with β-actin. N = 4 animals per age and genotype. (5.91 MB TIF) [file pone.0011464.s001.tif]

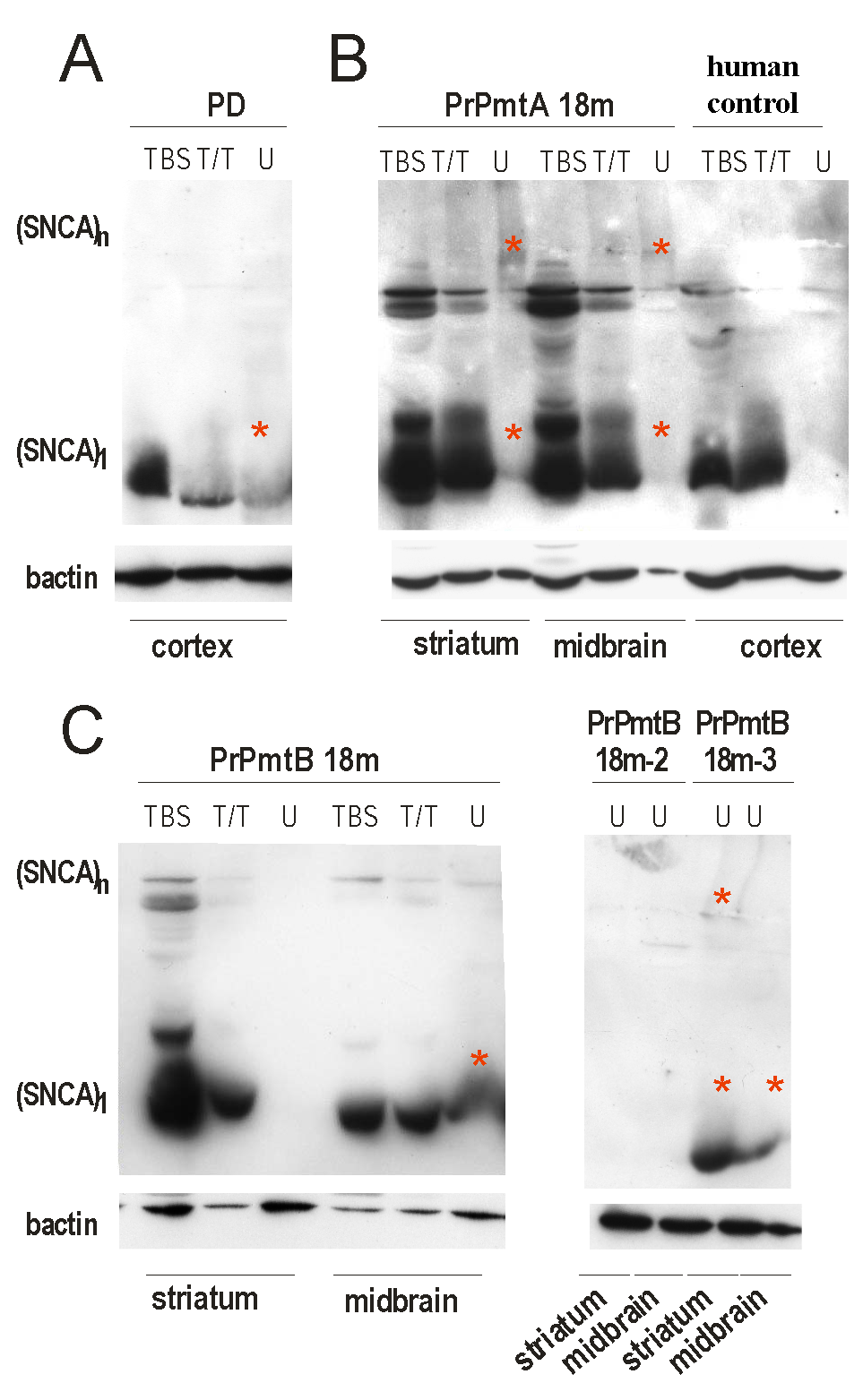

Supplement: Figure S2 — Insoluble alpha-synuclein in human PD and old A53T-SNCA brain. Tissue samples were subjected to sequential protein extraction as described in materials and methods, including cortex tissue (A) from a patient with Parkinson's disease (PD; BrainNet, Munich) as positive control and (B) from healthy human individual as negative control, together with samples of striatum and midbrain from (B) 18-month-old PrPmtA mice and (C) PrPmtB mice. The immunoblots were detected with an antibody recognizing the NAC fragment of human and mouse alpha-synuclein. Thus, signals in TBS-soluble fractions correspond to soluble alpha-synuclein, T/T signals correspond to membrane-bound alpha-synuclein and bands in the urea (U) fractions represents insoluble alpha-synuclein. (A) In the analyzed disease patient, alpha-synuclein was detected in the soluble (TBS), membrane-bound (T/T) and in the insoluble (U) extracts. (B) Comparable to signals seen in the PD patient, also the the insoluble fraction of PrPmtA nigrostriatal tissue contained monomeric alpha-synuclein and faint high molecular bands. In a healthy human control individual, alpha-synuclein signal was detected only in soluble and membrane-bound fraction, but not in the insoluble fraction of brain tissue. (C) A prominent insoluble monomeric (α-Syn)1 signal was detectable in midbrain of a 18-month-old PrPmtB transgenic mice (left panel), and (right panel) insoluble alpha-synuclein was found as high molecular forms (α -Syn)n in striatum and as monomeric species in striatum and midbrain of a second 18-month-old PrPmtB mouse, but not in a third analyzed mouse. Bands of insoluble anti-alpha-synuclein-immunoreactivity were highlighted with asterisks. Beta-actin was used as loading control. (4.54 MB TIF) [file pone.0011464.s002.tif]

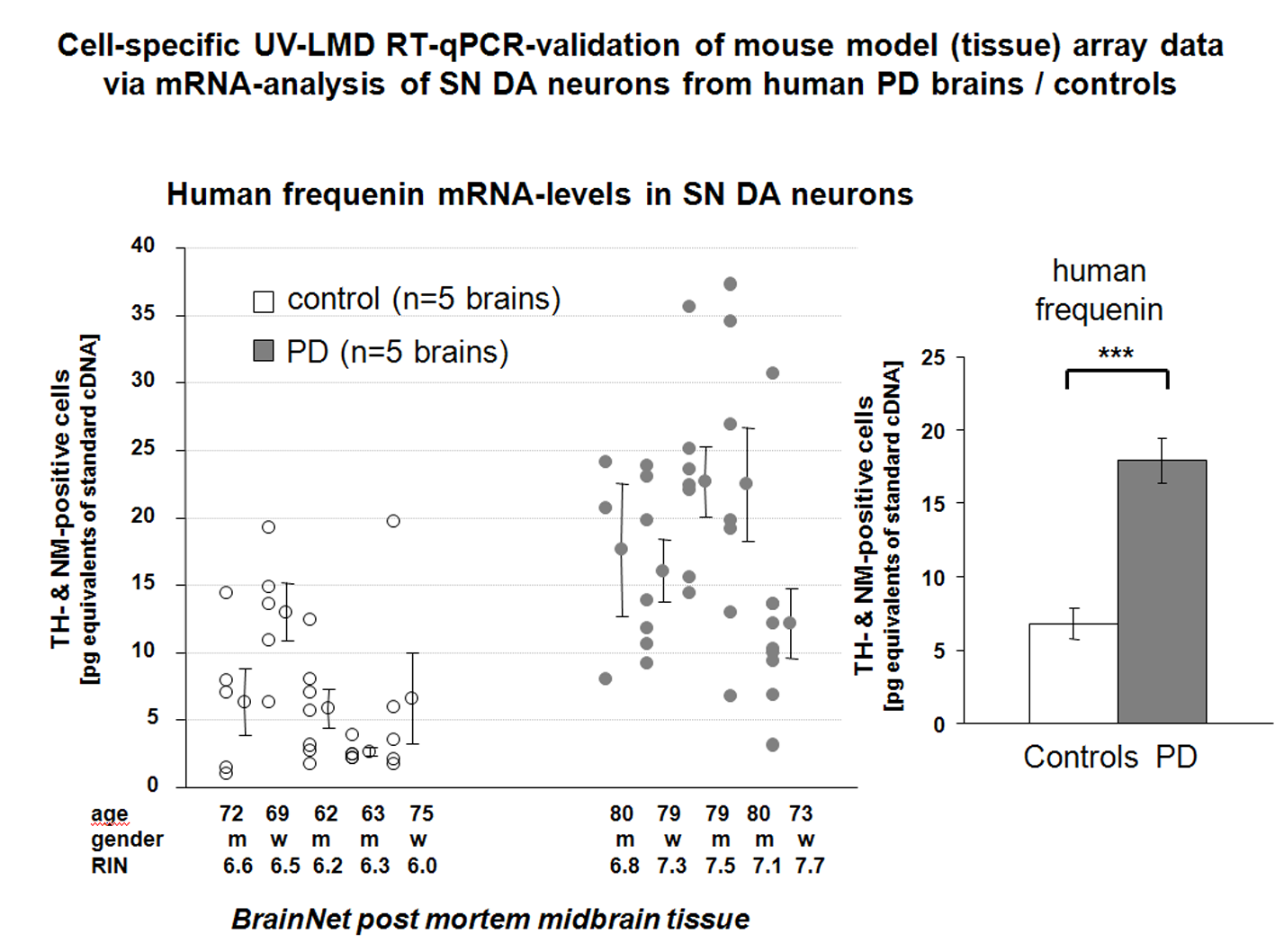

Supplement: Figure S3 — Altered frequenin mRNA expression of individual substantia nigra dopaminergic neuron from human PD post mortem midbrain via UV-LMD and qPCR. Neuromelanin-positive [NM(+)] neurons were isolated via UV-laser microdissection from cresylviolet-stained horizontal midbrain cryo-sections from PD and control brains (n = 5 each, provided from the German BrainNet). Left: Scatter plot of frequenin mRNA levels in PD and control brains. mRNA expression of each pool of 15 NM- and tyrosine hydroxylase- (TH-) positive SN DA neurons is given as pg-equivalents of total cDNA, derived from human SN tissue, per cell (determined via qPCR, standard curve quantification). Bars represent mean±SEM frequenin mRNA levels for all analyzed SN DA pools of each individual brain, (age, gender and RNA integrity number (RIN) for each brain is given at the bottom). Right: Mean mRNA level of frequenin was significantly higher in individual NM(+) SN DA neurons from PD brains compared to those of controls (PD: 17.89±1.54 pg, n = 33; controls 6.78±1.07, n = 27; p = 2×10∧−7). (5.21 MB TIF) [file pone.0011464.s003.tif]
